# A274 COVID-19 INFECTION RISK BETWEEN VACCINATED PATIENTS WITH ULCERATIVE COLITIS AND CROHN’S DISEASE : A RETROSPECTIVE COHORT STUDY IN TAIWAN

**DOI:** 10.1093/jcag/gwad061.274

**Published:** 2024-02-14

**Authors:** Y WU, J Chou

**Affiliations:** China Medical University Hospital, Taichung, Taiwan; China Medical University Hospital, Taichung, Taiwan

## Abstract

**Background:**

The effectiveness of coronavirus disease 2019 (COVID-19) vaccine against severe acute respiratory syndrome coronavirus 2 (SARS-CoV-2) in patients with inflammatory bowel disease (IBD) is well established, but it is not clear whether patients with ulcerative colitis (UC) and Crohn’s disease (CD) have different risks of COVID-19 infection after vaccination.

**Aims:**

The aim of this study was to compare the risk of COVID-19 infection after vaccination in patients with UC and CD. Moreover, we also investigated risk factors related to COVID-19 infection and collected data on any adverse events and disease symptoms.

**Methods:**

We conducted a retrospective cohort study in adult IBD patients who had received at least two doses of COVID-19 vaccination and compared the prevalence of infection between UC patients and CD patients using the medical records of China Medical University Hospital between 1 January 2020 and 31 March 2023. All data management and odds ratios calculations were performed using version 9.4 of SAS software (SAS Institute, Cary, NC, USA).

**Results:**

Total 169 IBD patients (96 with UC, 73 with CD) were included in this study. UC patients were older than CD patients (44.92±13.72 vs. 37.27±15.27 years, p=0.0008)(Table 1). A high proportion were male (IBD 70.41%; UC 65.63%, and CD 76.71%). Most (57.4%) received three doses of COVID-19 vaccines. Azathioprine, steroid, and mesalazine medication histories were different between the groups (pampersand:003C0.05). COVID-19 infection prevalence was 49.11% (UC 56.25%, CD 39.73%; p=0.0333) (Figure 1). Logistic regression analysis suggested UC patients had an odds ratio of 1.95 (95% CI=1.05-3.62) and adjusted odds ratio of 2.78 (95% CI=1.20-6.44) for COVID-19 infection compared to CD patients, indicating a 1.95-fold to 2.78-fold higher risk (Figure 2). There was a trend towards a decreasing risk of COVID-19 infection with increasing number of vaccine doses. Medication was not identified as a risk factor. (Figure 3)

**Conclusions:**

Our study identified a greater risk of COVID-19 infection in vaccinated patients with UC than those with CD. However, most patients who became infected with COVID-19 did not have severe symptoms and only one case was hospitalized. The mortality rate of COVID-19 in IBD patients is not specifically higher than the general population. Supporting the view that SARS-CoV-2 vaccination is effective in patients with IBD.

Odds ratios (95% confidence interval) for logistic regression model predicting COVID-19 infection among vaccinated IBD patients

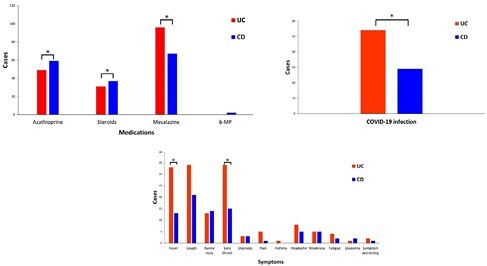

Figure 1. The medications used before COVID-19 infection between vaccinated patients with ulcerative colitis (UC) and Crohn’s disease (CD).

Figure2. The prevalence of COVID-19 infection between vaccinated patients with ulcerative colitis (UC) and Crohn's disease (CD).

Figure 3. The symptoms after COVID-19 infection between vaccinated patients with ulcerative colitis (UC) and Crohn's disease (CD).

**Funding Agencies:**

None

**Table:** 

	COVID-19	p-value	Adjusted Odds Ratio
UC	36	0.3618	0.3618
CD	17	0.1806	1.07

